# Resistance of *Wolbachia* to Trimethoprim: Insights into Genes Encoding Dihydrofolate Reductase, Thymidylate Synthase and Serine Hydroxymethyltransferase in the Rickettsiales

**DOI:** 10.3390/insects16010018

**Published:** 2024-12-28

**Authors:** Ann M. Fallon

**Affiliations:** Department of Entomology, University of Minnesota, St. Paul, MN 55108, USA; fallo002@umn.edu

**Keywords:** *Wolbachia*, dihydrofolate reductase, thymidylate synthase, trimethoprim, insect cell line, one-carbon transfer reactions, *folA*, *thyX*, *glyA*

## Abstract

Bacterial and eukaryotic dihydrofolate reductase (DHFR) enzymes are essential for DNA synthesis and are differentially sensitive to the competitive inhibitors trimethoprim and methotrexate. Unexpectedly, trimethoprim did not reduce *Wolbachia* abundance, and the *w*Stri DHFR homolog contained amino acid substitutions associated with trimethoprim resistance in *E. coli*. A phylogenetic tree showed good association of DHFR protein sequences with supergroup A and B assignments. In contrast, DHFR is not encoded by *w*Fol (supergroup E) and *w*Bm (supergroup D) or by genomes of the closely related genera *Anaplasma*, *Ehrlichia*, *Neorickettsia*, and possibly *Orientia*. In *E. coli* and humans, DHFR participates in a coupled reactions with the conventional thymidylate synthase (TS) encoded by *thyA* to produce the dTMP required for DNA synthesis. In contrast, *Wolbachia* and other Rickettsiales express the unconventional FAD-TS enzyme encoded by *thyX*, even when *folA* is present. The exclusive use of FAD-TS suggests that *Wolbachia* DHFR provides a supplementary rather than an essential function for de novo synthesis of dTMP, possibly reflecting the relative availability of, and competing demands for, FAD and NAD coenzymes in the diverse intracellular environments of its hosts. Whether encoded by *thyA* or *thyX*, TS produces dTMP by transferring a methyl group from methylene tetrahydrofolate to dUMP. In the Rickettsiales, serine hydroxymethyltransferase (SMHT), encoded by a conserved *glyA* gene, regenerates methylene tetrahydrofolate. Unlike *thyA*, *thyX* lacks a human counterpart and thus provides a potential target for the treatment of infections caused by pathogenic members of the Rickettsiales.

## Simple Summary

*Wolbachia* is an obligate intracellular bacterium common in insects and filarial worms. The elimination of *Wolbachia* using antibiotics provides a useful tool for investigating *Wolbachia*’s effects on host cells and exploring reproductive phenotypes such as cytoplasmic incompatibility associated with *Wolbachia* infection. I investigated whether the antibiotic trimethoprim, which has differential effects on the enzyme dihydrofolate reductase (DHFR) in bacterial and eukaryotic cells, could be used to eliminate the *Wolbachia* infection from an infected mosquito cell line. Surprisingly, the *Wolbachia* strain *w*Stri was resistant to trimethoprim, and its DHFR enzyme had amino acid substitutions associated with resistance in *E. coli*. However, further phylogenetic analysis revealed that some *Wolbachia* lack the *folA* gene encoding DHFR, suggesting that this gene is undergoing genomic streamlining and may not be essential to *Wolbachia* survival. In *E. coli* and in humans, DHFR participates in a coupled reaction with thymidylate synthase (TS) to produce dTMP, a key precursor in DNA synthesis. The absence of *folA* from some *Wolbachia* genomes led to an evaluation of TS and serine hydroxymethyltransferase (SMHT) in the Rickettsiales. All members of the Rickettsiales use the *thy*X-encoded FAD-TS rather than the conventional *thyA*-encoded TS, regardless of the presence of *folA*. The use of FAD-TS suggests that DHFR may not be essential in *Wolbachia*. Both TS enzymes transfer a methyl group from methylene tetrahydrofolate to dUMP; all Rickettsiales use SHMT encoded by the conserved *glyA* gene to restore the methyl group. Because the FAD-TS encoded by *thyX* lacks a human counterpart, it provides a potential target for treatment of infections caused by pathogenic members of the Rickettsiales.

## 1. Introduction

Antibiotics that have differential effects on eukaryotes and prokaryotes provide a potential tool to manipulate the growth of intracellular bacteria such as *Wolbachia* with minimal effects on their host cells. Prokaryotic dihydrofolate reductase (DHFR; E.C. 1.5.1.3) enzymes share key catalytic residues and folding patterns with eukaryotic homologs [1] but are differentially sensitive to competitive inhibitors. For example, the synthetic antifolate trimethoprim has 10^5^-fold higher affinity for prokaryotic relative to eukaryotic DHFR [2,3]. A casual blastp search showing that pandemic *Wolbachia* supergroup A and B genomes encode *folA* homologs of *E. coli* DHFR suggested that trimethoprim might be an alternative to rifampicin and tetracycline for removing *Wolbachia* infections from host cells.

Unexpectedly, the persistent supergroup B *Wolbachia* infection in C/*w*Stri1 cells was insensitive to relatively high trimethoprim concentrations that affected the growth of uninfected host cells. A bioinformatics-based analysis of *Wolbachia* DHFR proteins revealed well-conserved amino acid substitutions associated with trimethoprim resistance in *E. coli* [4] as well as additional amino acid substitutions that reflect supergroup assignments of *Wolbachia* A and B strains, for which abundant representatives are available. In contrast, *folA* was absent from some completed *Wolbachia* genomes, including *w*Fol (supergroup E, from the Collembolan, *Folsomia candida*) and *w*Bm (supergroup D, from the nematode *Brugia malayi*). More broadly among the Rickettsiales, DHFR proteins are not encoded by genomes of *Anaplasma*, *Ehrlichia*, *Neorickettsia*, and some but not all members of the genus *Rickettsia*.

To gain additional insight into the role of *Wolbachia* DHFR, I examined genes encoding serine hydroxymethyltransferase (SHMT) and thymidylate synthase (TS) in the Rickettsiales. Both enzymes, along with DHFR, participate in essential one-carbon transfers involved in folate metabolism. We have previously shown that SHMT, which is well conserved among the Rickettsiales, is one of the most abundant components of the *w*Stri proteome [5]. In humans and *E. coli*, the conventional *thyA*-encoded TS transfers a methyl group to dUMP, generating dTMP and dihydrofolate. DHFR converts the dihydrofolate back to tetrahydrofolate using NADPH as the cofactor. This cooperative interaction between DHFR and TS was long thought to be uniquely involved in the reductive methylation of dUMP to form dTMP.

In genomes that lack *folA*, an unconventional FAD-TS enzyme encoded by *thyX* produces dTMP by a distinct catalytic mechanism that does not involve DHFR [6]. In the Rickettsiales, TS is universally encoded by *thyX* rather than *thyA*, even in genomes that retain *folA*. Both TS enzymes use methylene tetrahydrofolate as the methyl donor in the conversion of dUMP to dTMP, and in all Rickettsiales, methylene tetrahydrofolate is regenerated by a conserved serine hydroxymethyltransferase (SHMT) encoded by *glyA*. These observations increase interest in metabolic reactions involving one-carbon transfers and the unique aspects of these processes as they relate to therapeutic applications in parasitic diseases [7]. In particular, *thyX* lacks a human homolog [8,9] and is a potential target for treatment of pathogenic members of the Rickettsiales [9], including tick- and insect-borne rickettsial diseases and filarial infections that depend on *Wolbachia*.

## 2. Materials and Methods

### 2.1. Cells and Culture Conditions

C7-10 cells are a wild type, standard cell line from the mosquito *Aedes albopictus* [10]. C/*w*Stri1 cells are a persistently infected derivative of C7-10 cells generated by infection with *w*Stri [11]. Cells were cultured in Eagle’s medium supplemented with non-essential amino acids, glutamine, vitamins, glucose, and antibiotics as described previously [12]. Heat-inactivated fetal bovine serum was used at a final concentration of 5%.

Trimethoprim was dissolved in DMSO at a final concentration of 50 mg/mL and stored frozen. Just before use, a 0.2 mg/mL solution was prepared in cell culture medium, and volumes of 0–80 μL were added to plates containing cells in 2 mL of culture medium. To monitor growth, cells were resuspended in the culture medium and diluted in isotonic saline, and duplicate counts were obtained from three replicate plates using a Coulter electronic cell counter. Flow cytometry was performed on an Attune NxT (Invitrogen) instrument with minor modifications relative to the EPICS instrument described previously [13]. Values represent duplicate samples from a single plate. For microscopy, cells were stained with a mixture of Syto13 and propidium iodide [14]. Photographs were taken with an Olympus 1 × 70 fluorescence microscope equipped with SPOT RT software version 3.2 (Diagnostic Instruments Inc., Sterling Heights, MI, USA).

### 2.2. Bioinformatics

Bioinformatics analyses were performed using blastp and Psi blast programs on the NCBI website (National Center for Biotechnology Information; Bethesda, MD, USA; https://www.ncbi.nlm.nih.gov/, accessed 11 Nov. 2024). Particular focus was placed on the well-established representative genomes of supergroup A strains *w*Mel, from *Drosophila melanogaster* (taxid 163164) and *w*Ri (taxid 66084), *w*Ha (taxid 1236909), and *w*Au (taxid 225364) from *Drosophila simulans*; supergroup B strains *w*No (taxid 1236908) from *Drosophila simulans*, *w*Stri (taxid 368602) from the planthopper *Laodelphax striatellus*, and *w*PipPEL (taxid 570417) from *Culex pipiens quinquefasciatus* mosquitoes; supergroup C strain *w*Ovol (taxid 77551) from the filarial worm *Onchocerca volvulus*; supergroup D strain *w*Bm (taxid 80849) from the filarial worm *Brugia malayi*; supergroup E strain *w*Fol (taxid 169402) from the springtail *Folsomia candida*; and supergroup F strain *w*Cle (taxid 246273) from the bedbug *Cimex lectularius*. The NCBI refseq_protein database (https://www.ncbi.nlm.nih.gov accessed 11 Nov. ember, 2024) was used to compare accessions across genera of Rickettsiales using the *w*Stri accession as the query. Note that it is not straightforward to search the genus “*Rickettsia*” because many organisms, including *Wolbachia pipientis*, which have been loosely assigned to Rickettsia, have been reclassified. Values for *Rickettsia sensu stricto* were approximated by searching the family “Rickettsiaceae” with exclusion of the genera *Anaplasma*, *Ehrlichia*, *Neorickettsia*, *Orientia*, and *Wolbachia*.

Phylograms produced on the Phylogeny.fr website (http://www.phylogeny.fr/index.cgi, Marseille, France; 11 Nov. 2024) using the “one click” mode with G-blocks disabled [15,16] were based on multiple sequence comparison by log-expectation (MUSCLE, version 3.8.31) [17]; phylogenies were constructed with PhyML (3.1/3.0 aLRT) and tree rendering with TreeDyn (198.3), as detailed on the website. Bootstrapping was based on likelihood ratio tests [18,19,20], and for production of figures, branches with less than 50% support were collapsed. Phylogenies show accession number, *Wolbachia* strain, and supergroup designation.

## 3. Results and Discussion

### 3.1. Dose Response of Uninfected Mosquito Cells to Trimethoprim

**Figure 1:**
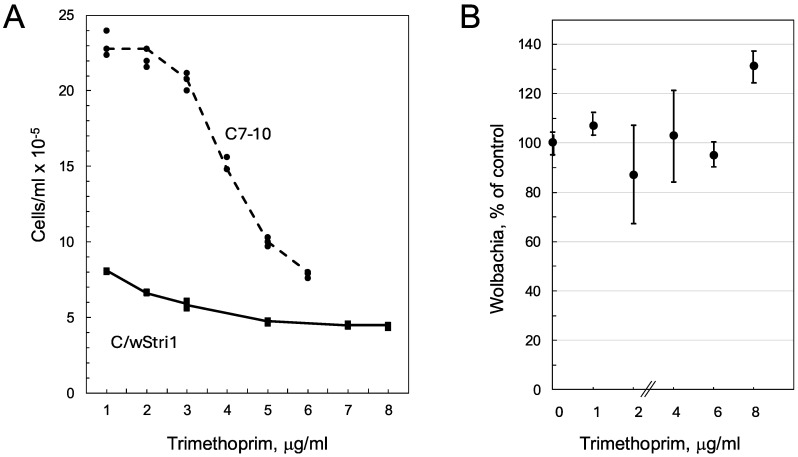
Effect of trimethoprim on cell growth and *Wolbachia* abundance. Panel (**A**): Control C7-10 *Aedes albopictus* cells and *Wolbachia*-infected C/*w*Stri1 cells. C7-10 cells were plated in 35 mm dishes at 2 × 10^5^ cells/plate in 2 mL of E-5 medium containing indicated concentrations of trimethoprim. On day 6, C7-10 cells reached confluency, and all plates were resuspended and counted in a Coulter electronic cell counter. C/wStri1 cells were diluted 5-fold and allowed to attach before addition of trimethoprim. Some individual data points are obscured by overlapping symbols. Panel (**B**). Duplicate samples from a single plate were assayed by flow cytometry. *Wolbachia* counts correspond to the lower left (Q3) quadrant as detailed previously (see figure 5, day 9 in [13]). Points indicate average values; bars indicate range.

**Figure 2:**
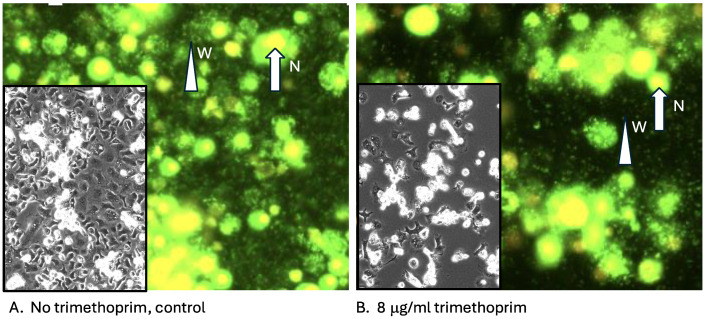
Microscopic appearance of *Wolbachia* in infected cells. Cells from Figure 1B were stained with a mixture of Syto13 and propidium iodide and photographed using fluorescence microscopy. Small particles marked W identify *Wolbachia* bacteria, which occur both as intracellular and extracellular particles. Overexposed, larger green staining identifies host cell nuclei. Grayscale insets show the distribution of adherent cells on plastic petri plates.

Trimethoprim is a synthetic diaminopyrimidine used to inhibit bacterial DHFR, which in clinical applications is often combined with sulfamethoxazole to reduce bacterial resistance. When used alone, the MIC (minimal inhibitory concentration) of trimethoprim for *E. coli* is 0.2 μg/mL [21]. To establish the sensitivity of *Aedes albopictus* mosquito cells to trimethoprim, wild-type C7-10 cells seeded at 2 × 10^5^ cells per plate were grown in the presence of the drug until they reached confluency (4.6 × 10^6^ cells/plate). Growth was inhibited by 50% at 4–5 μg of trimethoprim per ml (Figure 1A). C/*w*Stri1 cells, which are persistently infected with *Wolbachia* strain *w*Stri, are larger than C7-10 cells, have a reduced plating efficiency, and a slower doubling time. Over a period during which C7-10 cells increased 20-fold in number, C/*w*Stri1 cells barely doubled. Trimethoprim inhibited the growth of both cell lines but had a negligible impact on total *Wolbachia* abundance as determined by flow cytometry (Figure 1B) and microscopic comparison of untreated control C/*w*Stri1 cells relative to cells treated with 8 μg/mL trimethoprim. *Wolbachia* were visible both within cells and as freely floating extracellular particles (Figure 2, white triangles, W). Note that overexposure of Syto13-stained nuclei (Figure 2, white arrow labeled N) was required to visualize extracellular *Wolbachia*. As shown in the phase contrast insets (Figure 2), trimethoprim increased the tendency of cells to aggregate and reduced overall cell density.

### 3.2. Wolbachia DHFR Contains Amino Acids Associated with Trimethoprim Resistance

**Figure 3:**
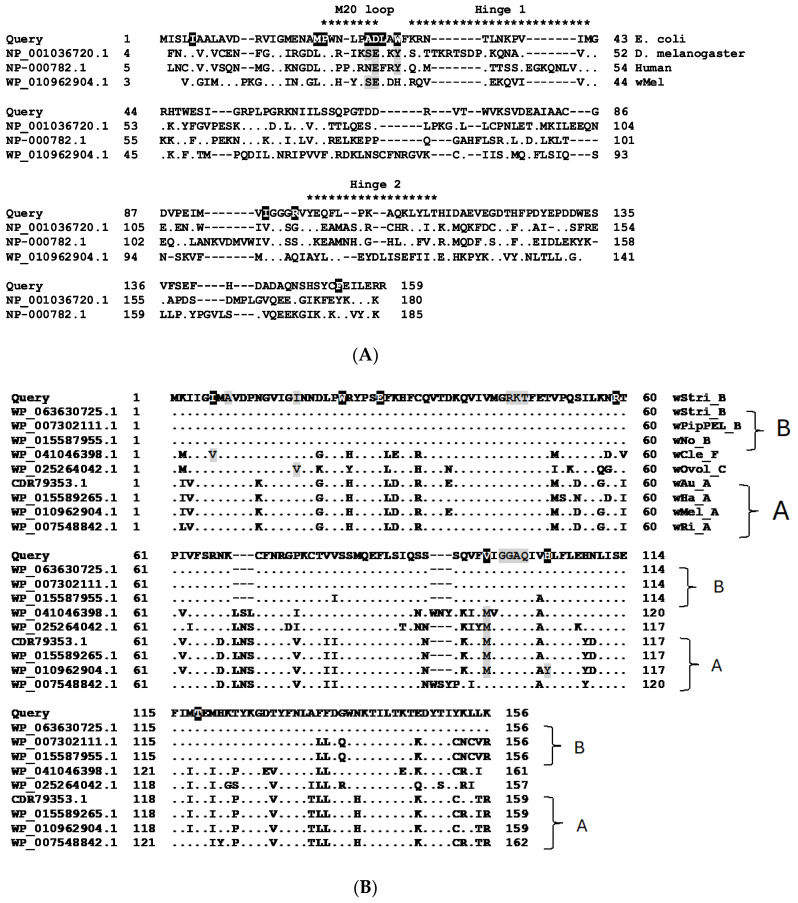
Comparison of DHFR proteins. (**A**) Alignment of *E. coli* (query, NP414590.1), *D. melanogaster*, human, and *w*Mel DHFR proteins. Substitutions at residues in white font on a black background have been associated with trimethoprim resistance in *E. coli*. Gray shading indicates specific substitutions associated with resistance that occur in *D*. *melanogaster*, human, and *w*Mel proteins. (**B**) Alignment of DHFR homologs from selected *Wolbachia* strains for which genomes are complete or nearly complete. In these alignments, white font with black shading identifies residues that interact with folate, and gray shading identifies residues that interact with NADP. Note the absence of a homolog in the *w*Bm supergroup D and *w*Fol supergroup E.

The DHFR enzyme encompasses three structural regions important for enzyme function: the M-20 loop, spanning *E. coli* residues P21 to D27; Hinge 1, extending from F31 to M42; and Hinge 2, from Y100 through T113 [1]. Manna et al. [4] correlate trimethoprim resistance with 10 different amino acid substitutions (Figure 3A, white text with black shading in the *E. coli* sequence shows the wild-type residue): I15F, M20I, P21Q/L, A26T/V/S, D27/E, L28R, W30R/G/C/Y, I94L, R89P, F153V/L. When aligned, *E. coli* DHFR, human, *Drosophila melanogaster*, and its *Wolbachia* endosymbiont *w*Mel shared 26–29% amino acid identity. Potential resistance-associated substitutions A26S and D27E occur in both the *D. melanogaster* and the bacterial *w*Mel homologs, while D27E and W30Y occur in *Drosophila* and human proteins (Figure 3A, shaded gray). Overall, only 14 amino acid residues dispersed over the length of the protein were universally conserved among these four divergent DHFR homologs.

### 3.3. Some Wolbachia Genomes Do Not Encode DHFR Proteins

Identities among a representative group of *Wolbachia* DHFR proteins (supergroup A: wMel, wRi, wHa, wAu; supergroup B, wNo, wStri, wPipPel; supergroup C, wOvol; supergroup D, wBm; supergroup E, wFol; supergroup F, wCle) were at least 74%, with nearly complete conservation of sites that interact with folate (Figure 3B, white font with black shading) and NADPH (Figure 3B, gray shading). Among these key residues, the few that differ are shaded gray within the alignment. Supergroups A and B clustered into distinct patterns, but unexpectedly, *w*Bm and *w*Fol from supergroups D and F lacked annotated DHFR proteins. The absence of DHFR in other bacteria [6,22] and the streamlined nature of *Wolbachia* genomes suggest that *folA* may not be an essential gene in *Wolbachia*. To examine more closely the distribution of DHFR proteins among the order Rickettsiales (taxid 766), individual genera excluding *Wolbachia* (taxid 953) were queried using the refseq_proteins database (Table 1). In the genus *Rickettsia*, 42 *w*Stri DHFR homologs were recovered, including members of the spotted fever and bellii groups, but homologs were missing from *R. prowazekii* and *R. typhi* in the typhus group. Likewise, *folA* homologs were absent from *Anaplasma*, *Ehrlichia*, and *Neorickettsia* queried individually as separate genera. In the NCBI nr database, a single accession for *Orientia tsutsugamushi* (SPR13141) lacks the first 42 DHFR N-terminal residues.

### 3.4. An FAD-Dependent Thymidylate Synthase Is Encoded by thyX in the Rickettsiales

In both *E. coli* and humans, the cooperative interaction between DHFR and an NADPH-dependent TS (E.C. 2.1.1.45), encoded by *thyA*, catalyzes the reductive methylation of dUMP to form dTMP and dihydrofolate with regeneration of tetrahydrofolate by DHFR. Before large numbers of microbial genomes became available, it was thought that *folA* was essential. More recent confirmation that *folA* is absent from about 30% of bacterial genomes [6,8] led to the discovery of a second, nonconventional FAD-TS (E.C. 2.1.1.148), encoded by *thyX*, believed to have evolved independently of *thyA* [8]. Like *thyA*-encoded TS, however, FAD-TS uses methylene tetrahydrofolate as the methyl donor, but it differs in using FADH_2_ as the reductant. Blastp and PSI-blast searches with *E. coli thyA*-encoded TS (WP_001308885.1) failed to uncover homologs in the Rickettsiales (taxid 766), even in those strains where *folA* is present.

**Figure 4:**
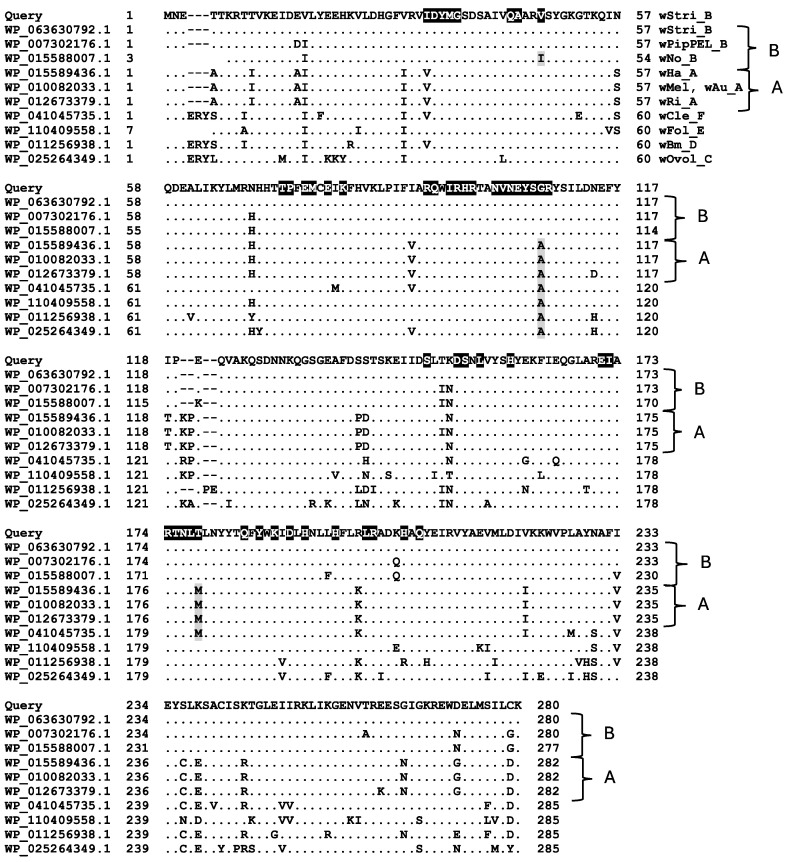
Alignment of *Wolbachia* TS proteins encoded by *thyX*. Note that homologs are present in in *w*Bm supergroup D and *w*Fol supergroup E. Residues in white font on a black background are NCBI conserved domains related to FAD binding, nucleotide binding, and tetramer interface. Letters following *Wolbachia* designations indicate supergroups. Gray-shaded residues designate differences in key conserved sites.

**Figure 5:**
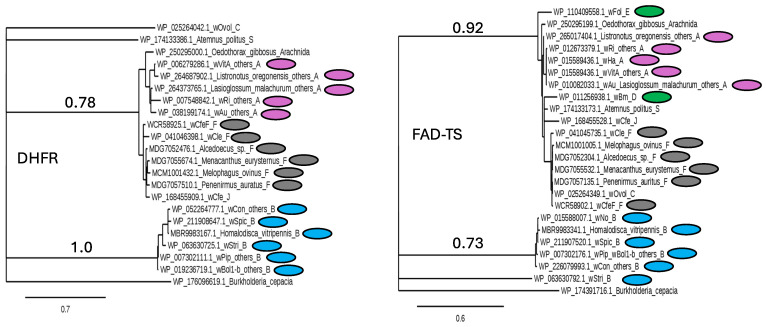
Phylogeny of *Wolbachia* DHFR and TS proteins shows correlation with supergroup assignments. Trees were produced on the phylogeny.fr website as described in the Materials and Methods section. Colored ovals indicate *Wolbachia* supergroups A, magenta; B, blue; and F, gray. *Wolbachia* that lack *folA* are indicated in green in the FAD-TS panel at right.

A PSI-blast search with FAD-TS encoded by *Helicobacter pylori thyX* recovered a homolog in *Orientia tsutsugamushi* (WP_045915720.1), and further searches showed that the *thyX* encodes FAD-TS in all genera within the Rickettsiales. An alignment shows that FAD-TS has 85% or greater identity among representative *Wolbachia* genomes, including *w*Fol and *w*Bm, which lack *folA*. The alignment agrees with supergroup assignments and shows nearly complete conservation of residues involved in FAD binding, nucleotide binding, and the tetramer interface (Figure 4). Trees showing the evolution of *Wolbachia* DHFR and FAD-TS proteins (Figure 5) relative to the sequence from the outgroup beta proteobacterium *Burkholderia cepacia* suggest that genes encoding both DHFR and FAD-TS (in the FAD-TS panel, note the green symbols for *w*Fol and *w*Bm) are evolving at similar rates, and that protein sequences are consistent with supergroup designations. Although larger-scale examinations did not uncover unusual features among these proteins, it might be of interest to explore genomes that lack a full-length *folA* for traces of related coding sequences. Finally, the universal presence of *thyX* makes it difficult to evaluate the importance of the amino acid substitutions associated with trimethoprim resistance in *Wolbachia* DHFR homologs given that the role, if any, of *Wolbachia* DHFR differs from that in *E. coli* and other organisms for which trimethoprim is effective.

**Figure 6:**
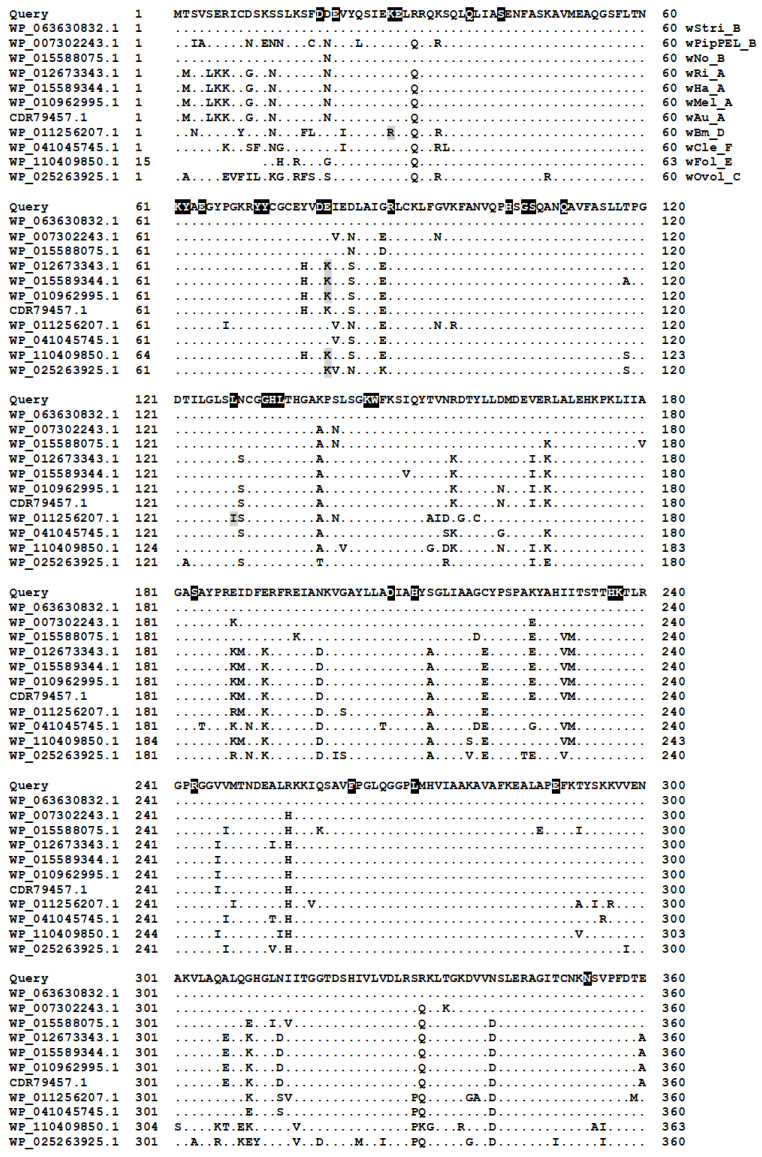

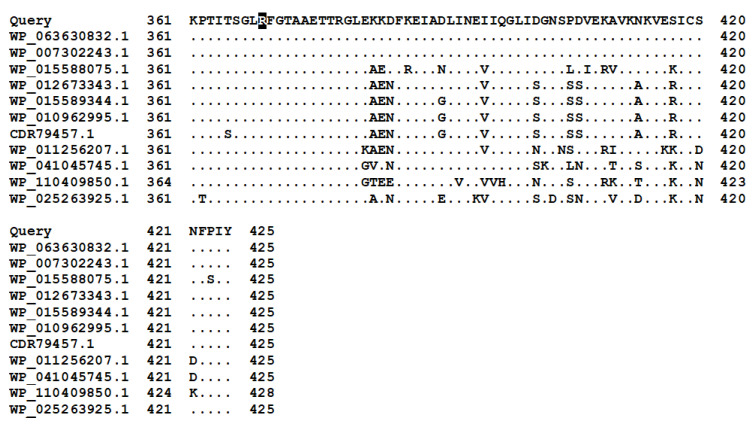
Alignment of SHMT proteins. Residues in white font on a black background identify residues as-sociated with the active site, glycine pyridoxal phosphate binding, folate binding, and dimer inter-face. Gray-shaded residues designate differences in key conserved sites.

Regardless of whether TS is encoded by *thyA* or *thyX*, methylene tetrahydrofolate, which donates a methyl group to dUMP, needs to be restored. An important source of methyl groups in the folate pathway is serine hydroxymethyltransferase (SMHT; E.C. 2.1.2.1; WP_063630832.1, encoded by *glyA*), one of the most abundant enzymes in the wStri proteome [5]. Like thyX, *glyA* is well conserved in *Wolbachia* (Figure 6), and among the Rickettsiales, a systematic search of the refseq_proteins databases shows that the number of annotated SHMT homologs follows the distribution of FAD-TS and differs from DHFR (Table 1). This comparison confirms the underrepresentation of DHFR annotations relative to those of FAD-TS and SHMT in Rickettsiales genomes.

Relative to the classical TS encoded by *thyA*, the FAD-TS encoded by *thyX* is poorly understood, but elucidation of its distinct catalytic mechanisms provides important insights into the size, composition, and evolution of prokaryotic genomes [6]. In bacteria that encode *folA* and both *thyA* and *thyX*, evidence suggests that the two TS enzymes may not be functionally equivalent. In *Corynebacterium glutamicum*, *thyA* is thought to be essential, while in *Mycobacterium tuberculosis*, mutational studies define *thyX* as essential [22]. Aside from providing new insight into the folate cycle, studies with *M. tuberculosis* suggest that *thyX*, which has no human homolog, is a potential target for therapeutic drugs. Because the Rickettsiales include pathogenic species [23,24], it will be of interest to evaluate their susceptibility to drugs that specifically target FAD-TS without affecting the classical TS used in humans. Among the Rickettsiales, the universal presence of *thyX* rather than *thyA* and the partial absence of *folA* suggests that DHFR provides a supplementary rather than an essential function in the generation of dTMP. The retention of *folA* in some but not all *Wolbachia* genomes suggests that this gene is subject to genomic streamlining, while its retention in some genomes may reflect the relative availability of, and competing demands for, FAD and NAD-containing coenzymes in the intracellular environment of invertebrate host cells. Finally, SHMT is particularly abundant in the *w*Stri proteome [5], plays a key role in the interconversion of serine and glycine using tetrahydrofolate as the one-carbon carrier, and itself is being evaluated as an antibacterial target [25,26].

## 4. Conclusions

This analysis shows that the *Wolbachia* strain *w*Stri, which grows particularly well in a mosquito cell line, is insensitive to trimethoprim. These observations are consistent with earlier studies showing that co-trimoxazole, which contains trimethoprim, is ineffective against strain *w*AalbB in Aa23 cells [27]. Bioinformatic exploration indicates that the *folA* gene, which encodes the DHFR protein targeted by trimethoprim, is absent from many *Wolbachia* strains. Moreover, regardless of the presence of *folA*, all members of the Rickettsiales produce dTMP using the FAD-TS encoded by *thyX*. The absence of a conventional *thyA*-encoded TS, which requires DHFR in the production of dTMP, suggests that *folA* may be undergoing genomic streamlining and that trimethoprim resistance may be universal among the *Wolbachia*. Although direct experimental evidence remains to be extended beyond *w*Stri and *w*AlbB, these results suggest that trimethoprim is an unlikely candidate for eliminating *Wolbachia* infections and that inactivation of *thyX*-encoded TS may provide a more effective means of interfering with the production of dTMP in the Rickettsiales.

## Figures and Tables

**Table 1:** Distribution of protein accessions corresponding to genes encoding DHFR, FAD-TS, and SHMT involved in synthesis of dTMP. Details of the search (11 Nov. 2024) are described in the Materials and Methods section. The column labeled Rickettsiales indicates the total number of entries for the order. Sums at right are based on the total number of genus entries in each row.

Taxon	Rickettsiales	*Anaplasma*	*Ehrlichia*	*Neorickettsia*	*Orientia*	*Rickettsia*	*Wolbachia*	Sum
NCBI taxid	766	768	943	33,993	69474	775	953	
gene, query	accessions							
*folA*, WP_063630725.1	149	0	0	0	0	42	106	148
*thyX*, WP_063630792.1	285	17	10	5	14	84	134	264
*glyA*, WP_063630832.1	341	21	14	5	16	96	169	321

## Data Availability

Upon request.
